# Long-term follow-up outcomes for patients undergoing primary total hip arthroplasty with uncemented versus cemented femoral components: a retrospective observational study with a 5-year minimum follow-up

**DOI:** 10.1186/s13018-019-1415-3

**Published:** 2019-11-15

**Authors:** Tiejian Liu, Xiaoxiao Hua, Weiguang Yu, Jinluan Lin, Mingdong Zhao, Jun Liu, Xianshang Zeng

**Affiliations:** 1 Department of Neurosurgery, The Third Affiliated Hospital of Southern Medical University, No.183, Zhongshan avenue west, Tianhe District, Guangzhou, 510630 China; 2 Department of Anesthesiology, The Guanghua School of Stomatology, Hospital of Stomatology, Sun Yat-sen University, No. 56, Lingyuan West Road, Guangzhou, 510055 China; 3 Department of Orthopaedics, The First Affiliated Hospital, Sun Yat-sen University, No. 58, Zhongshan 2nd Road, Yuexiu District, Guangzhou, 510080 China; 4 Department of Orthopaedics, The Affiliated Hospital of Fujian Medical University, Chazhong Road No. 20, Taijiang District, Fuzhou, 350005 Fujian China; 5 Department of Orthopaedics, Jinshan Hospital, Fudan University, Longhang Road No. 1508, Jinshan District, Shanghai City, 201508 China; 6 Department of Gynaecology and Obstetrics, The First Affiliated Hospital, Sun Yat-sen University, No. 58, Zhongshan 2nd Road, Yuexiu District, Guangzhou, 510080 China

**Keywords:** Uncemented, Cemented, Total hip arthroplasty, Femoral neck fracture, Complication

## Abstract

**Background:**

This retrospective analysis compared the long-term outcomes for patients with a femoral neck fracture (AO/OTA type 31B) treated with a primary unilateral total hip arthroplasty with uncemented or cemented femoral components (UTHA or CTHA, respectively).

**Methods:**

We conducted a retrospective cohort study using the South China Hip Arthroplasty Database. We identified 422 patients with femoral neck fracture (AO/OTA type 31B) who were previously treated with primary unilateral UTHA or CTHA between 2007 and 2015, with follow-up until 2019. Follow-up occurred 1, 3, 6 and 12 months postoperatively and yearly thereafter. The primary outcome was the Harris hip score (HHS). The secondary outcome was the orthopaedic complication rate.

**Results:**

In total, 324 patients (UTHA *n* = 160, mean age 68.61 ± 7.49 years; CTHA *n* = 164, mean age 68.75 ± 7.04 years) were evaluated for study eligibility. The median follow-up was 73.3 months (range, 11.6–89.2 months). At the final follow-up, HHS was 74.09 ± 6.23 vs 79.01 ± 10.21 (UTHA vs CTHA, *p* = 0.012). Significant differences were detected in the incidence of prosthetic revision, loosening, and periprosthetic fracture between the UTHA and CTHA groups (7.5% for UTHA vs 1.8% for CTHA, *p* = 0.015; 17.5% for UTHA vs 8.5% for CTHA, *p* = 0.016; 11.9% for UTHA vs 4.9% for CTHA, *p* = 0.021, respectively).

**Conclusion:**

In this setting, CTHA demonstrated superiority to UTHA by improving functional outcomes and decreasing complication rates.

## Background

Femoral neck fractures potentially lead to catastrophic physical and functional impairment [1–4]. The demand for total hip arthroplasty (THA), which is an exceedingly successful surgery for femoral neck fractures, has gradually climbed in the elderly [1] and could increase by 174% by 2030 [5]. The increase in demand may also be contributing to the escalated implant-related failure rate. Total hip arthroplasty with an uncemented or cemented femoral component (UTHA or CTHA) is a relatively common procedure for the treatment of femoral neck fractures [6–8]. Prior studies [6, 9, 10] have shown perioperative benefits of CTHA over UTHA are fewer complications and shorter recovery times, but these findings were based on studies with small sample sizes. Nevertheless, there remain concerns that longer-term outcomes of UTHA may not be as robust as those of CTHA, that UTHA increases the need for early revision (< 5 years) and that UTHA requires unnecessary interventions to maintain normal hip function [8, 11]. The risks of early revision and reintervention, as well as the excessive expense associated with these supplementary procedures, could offset the primary benefit obtained from UTHA [12, 13]. Furthermore, highly selective characteristics of the studied individuals tend to exist in previous studies [6, 12, 14]. Hence, the findings in those studies could fail to reflect the actual situation.

To date, there is no direct comparison regarding long-term outcomes following UTHA or CTHA in the Asian population. Furthermore, there remain limited data regarding long-term reinterventions for problems following UTHA or CTHA, such as prosthetic loosening and periprosthetic fracture. In the present study, we used data from the South China Hip Arthroplasty Database to compare long-term outcomes for patients with femoral neck fracture (AO/OTA type 31B) treated with primary UTHA or CTHA.

## Materials and methods

### Study population

We performed a retrospective analysis with an initial study cohort that included 422 individuals with femoral neck fracture (AO/OTA type 31B) from the South China Hip Arthroplasty Database who were previously treated with primary unilateral UTHA or CTHA between June 2006 and December 2017. Two cohorts were identified using the procedural code for primary THA (9 81.51), according to the International Classification of Diseases, 9th Revision, Clinical Modification (ICD-9-CM) [5]. THA was performed by the same medical team using a direct anterior approach (DAA) [15]. Perioperative management was similar between groups, as previously reported [6, 8, 16]. The inclusion criteria were as follows: patients aged ≥ 60 years old; patients who sustained femoral neck fractures (AO/OTA type 31B) confirmed with antero-posterior (AP) and lateral radiographs or with CT/MRI and underwent an initial unilateral UTHA or CTHA (uncemented or cemented femoral component: Stryker Orthopaedics, Mahwah, SM, USA; acetabular component: standard-device, Stryker, Mahwah, NJ); and patients who were ambulatory prior to the fracture without an aid, such as a cane or a walker. The main exclusion criteria included scanty clinical data, revision arthroplasty, poly-trauma (e.g. ipsilateral or contralateral fractures of the foot, ankle, knee or femur), active infection around the hip, abnormal gait, osteomalacia, Paget’s disease, rheumatoid arthritis [17], marked bone loss, planned surgery, pathological fractures, dyspraxia, inability to follow instruction, loss to follow-up, malignant tumour, injury severity score (ISS) ≥ 10, presence of severe medical disease (e.g. poor long-term compliance with antihypertensive treatment and diffuse intravascular coagulation), body mass index (BMI) > 35 kg/m^2^, severe cardiac/respiratory impairment [18], mental illness preventing follow-up, vascular cognitive impairment (VCI) and dementia [19] and an American Society of Anesthesiologists (ASA) score of IV or V.

### Method of assessment

The primary outcome was the HHS, which has scores ranging from 0 to 100 points, with higher scores indicating better function, and contains subscales for pain, function, deformity and range of motion. The HHS was assessed at 1, 3, 6 and 12 months postoperatively and yearly thereafter. The secondary outcome was the orthopaedic complication rate. Patient-related complications included prosthetic loosening, prosthetic revision, periprosthetic fracture, dislocation, femur shaft fracture, infection around the prosthesis, intolerable hip pain, lower limb shortening (> 1.5 cm) and heterotopic ossification.

### Statistical analysis

The differences in demographic data and main outcome measures for the two cohorts were assessed. In the primary analysis, follow-up was calculated from the date of primary THA to the date of either death or the last documented follow-up, whichever came first. Revision was defined as the removal of the prosthesis or exchange of a part or the whole prosthesis. Loosening and implant failure were defined in accordance with the previous reports [1, 20]. Heterotopic ossification was assessed per the Brooker classification system [21]. Continuous variables and categorical data were analysed using independent *t* tests and chi-squared tests, respectively. The two-tailed level of significance was set at *p* < 0.05 for all statistical comparisons. All analyses were conducted using SPSS software, version 24 (IBM Corp, Armonk, NY).

## Results

In total, 324 patients had baseline data available from January 2007 to January 2015 (UTHA: *n* = 160, mean age, 68.61 ± 7.49 years; CTHA: *n* = 164, mean age, 68.75 ± 7.04 years). At the time of analysis, the median follow-up was 73.16 months (range 13.35–84.88 months) for UTHA and 73.25 months (range 14.21–86.43 months) for CTHA. The baseline data are summarised in Table 1.

**Table 1:** Patient demographics and outcomes

Variable	UTHA^a^ (*n* = 160)	CTHA^b^ (*n* = 164)	*p* value
Sex, M/F	97/63	92/72	0.409*^c^
Age, years	68.61 ± 7.49	68.75 ± 7.04	0.215*^d^
BMI, kg/m^2^	27.11 ± 7.75	27.36 ± 8.12	0.322*^d^
BMD	− 3.59 ± 0.45	− 3.64 ± 0.37	0.176*^d^
Side, left/right	86/74	81/83	0.432*^c^
Comorbidities, *n*%			0.296*^e^
Hypertension	33 (20.6)	29 (17.7)	
Diabetes mellitus	37 (23.1)	38 (23.2)	
Cardiopathy	15 (9.4)	17 (10.4)	
Hypertension and diabetes mellitus	22 (13.8)	25 (15.2)	
Pulmonary	24 (15.0)	26 (15.8)	
Cerebrovascular accident	16 (10.0)	20 (12.2)	
Anaemia	11 (6.9)	15 (9.1)	
Mechanism of injury, *n*%			0.454*^e^
Traffic-related injury	35 (21.8)	38 (23.1)	
Injury by falling	89 (55.6)	96 (58.5)	
Tamp injury	36 (22.6)	30 (18.4)	
ASA Index, *n*%			0.609*^e^
I	45 (28.1)	47 (28.6)	
II	76 (47.5)	83 (50.6)	
III	39 (24.4)	34 (20.8)	
Preoperative HHS	57.82 ± 16.48	57.64 ± 15.88	0.231*^d^
Follow-up period (months)	73.16 ± 11.72	73.25 ± 13.58	0.069*^d^

*UTHA* uncemented femoral component total hip arthroplasty, *CTHA* cemented femoral component total hip arthroplasty, *HHS* Harris hip score, *ASA* American Society of Anesthesiologists, *BMI* body mass index, *BMD* bone mineral density

*No statistically significant values

^a,b^Uncemented or cemented femoral component: Stryker Orthopaedics, Mahwah, NJ, USA; acetabular component: standard-device, Stryker Orthopaedics, Mahwah, NJ, USA

^c^Analysed using the chi-square test

^d^Analysed using an independent-samples *t* test

^e^Analysed using the Mann-Whitney test

### Primary outcome

The HHS before and after treatment are summarised in Table 2. Significant improvements in the HHS after surgery were detected in the two groups. Approximately, 93% of the patients with femoral neck fracture (AO/OTA type 31B) treated with primary UTHA or CTHA had a satisfactory HHS at the final follow-up. In the first or third month postoperatively, a between-group significant difference was not observed. From the third month postoperatively to the final follow-up, it appears that CTHA yielded better functional outcomes than UTHA.

**Table 2:** Long-term follow-up: postoperative functional outcomes

Month(s)	UTHA^a^ (*n* = 160)	CTHA^b^ (*n* = 164)	*p* value
At 1	79.14 ± 8.23	79.25 ± 7.59	0.236
At 3	87.64 ± 7.52	87.77 ± 7.63	0.062
At 6	89.14 ± 11.92	91.45 ± 9.57	0.041*
At 12	88.75 ± 8.06	90.48 ± 8.91	0.043*
At 13	88.11 ± 8.47	90.42 ± 9.02	0.040*
At 15	87.54 ± 10.21	89.78 ± 9.83	0.042*
At 18	86.38 ± 11.25	88.74 ± 8.32	0.038*
At 24	85.56 ± 11.79	88.62 ± 9.01	0.036*
At 25	85.29 ± 12.33	87.47 ± 10.58	0.042*
At 27	84.54 ± 11.22	86.85 ± 12.15	0.043*
At 30	82.63 ± 12.16	85.68 ± 13.72	0.037*
At 36	81.66 ± 10.27	84.75 ± 9.75	0.036*
At 37	80.11 ± 13.39	84.56 ± 11.47	0.032*
At 39	78.76 ± 13.27	84.56 ± 12.53	0.029*
At 42	78.16 ± 14.60	83.17 ± 13.29	0.030*
At 48	77.13 ± 13.46	82.33 ± 12.02	0.032*
At 49	76.12 ± 12.16	82.16 ± 13.10	0.027*
At 51	75.31 ± 11.10	81.57 ± 10.39	0.014*
At 54	76.65 ± 10.70	81.10 ± 8.02	0.011*
At 60	75.92 ± 8.47	80.17 ± 7.06	0.013*
At final follow-up	74.09 ± 6.23	79.01 ± 10.21	0.012*

*UTHA* uncemented femoral component total hip arthroplasty, *CTHA* cemented femoral component total hip arthroplasty

*Statistically significant values

^a,b^Uncemented or cemented femoral component: Stryker Orthopaedics, Mahwah, NJ, USA; acetabular component: standard-device, Stryker Orthopaedics, Mahwah, NJ, USA

### Secondary outcome

Thirty-six complications in 42 UTHA-treated patients versus 28 complications in 34 CTHA-treated patients were observed. Of the 36 complications in the UTHA-treated cohort, 12 (7.5%) required prosthetic revision, 28 (17.5%) were from prosthetic loosening, 19 (11.9%) were from periprosthetic fracture and 27 (16.9%) were intolerable hip pain. Of the 28 complications in the CTHA-treated cohort, 3 (1.8%) were from prosthetic revision, 14 (8.5%) were from prosthetic loosening, 8 (4.9%) were from periprosthetic fracture and 22 (13.4%) were intolerable hip pain (Table 3). A significant difference was detected between groups in the revision rate at the final follow-up (7.5% for UTHA vs 1.8% for CTHA, *p* = 0.015). Approximately 82.1% of revisions for UTHAs were attributed to periprosthetic fractures compared with 73.5% of revisions for CTHAs (*p* = 0.034). Periprosthetic loosening was a more frequent reason for revision among UTHAs than among CTHAs (*p* = 0.016), which cannot be treated conservatively.

**Table 3:** Long-term follow-up: prosthesis-related complications

Variable, *n*%	UTHA^a^ (*n* = 160)	CTHA^b^ (*n* = 164)	*p* value
Prosthetic revision	12 (7.5)	3 (1.8)	0.015*^c^
Prosthetic loosening	28 (17.5)	14 (8.5)	0.016*^c^
Periprosthetic fracture	19 (11.9)	8 (4.9)	0.021*^c^
Dislocation	11 (6.9)	5 (3.0)	0.112^c^
Femur shaft fracture	6 (3.8)	5 (3.0)	0.728^c^
Infection around the prosthesis	2 (1.3)	2 (1.2)	1.000^c^
Intolerable hip pain	27 (16.9)	22 (13.4)	0.385^c^
Lower limb shortening (> 1.5 cm)	10 (6.3)	6 (3.7)	0.282^c^
Heterotopic ossification	23 (14.4)	24 (14.6)	0.947^c^

*UTHA* uncemented femoral component total hip arthroplasty, *CTHA* cemented femoral component total hip arthroplasty

*Statistically significant values

^a,b^Uncemented or cemented femoral component: Stryker Orthopaedics, Mahwah, NJ, USA; acetabular component: standard-device, Stryker Orthopaedics, Mahwah, NJ, USA

^c^Analysed using the chi-square test

## Discussion

Our analysis provides evidence that CTHA was superior to UTHA because of the significantly increased HHS and distinctly reduced orthopaedic complication rate associated with CTHA in an Asian population with femoral neck fracture (AO/OTA type 31B). Moreover, the present analysis was conducted with data from a cohort representative of the “real world” practice.

In several randomised controlled trials [6, 12, 14], UTHA tended to be associated with a higher prosthetic revision rate and moderately lower HHS in comparison with those of CTHA. Given this, the application of CTHA in individuals with femoral neck fractures could have a significant influence on postoperative complications. When analysing the impact of CTHA on clinical complications, we failed to detect an increased risk for unfavourable outcomes, particularly prosthetic revision and loosening. Furthermore, previous studies [6, 9, 22] observed a distinctly low risk for CTHA-related complications. Although CTHA improves clinical outcomes, one safety concern is that interference with the cemented femoral component could promote the destabilisation of bone and cement components and thus increase the risk for complications, such as prosthetic loosening, prosthetic revision, periprosthetic fracture, dislocation, femur shaft fracture, hip pain, lower limb shortening and heterotopic ossification [10, 23]. This is of specific concern, as undergoing a THA has been considered a potential risk factor for morbidity and mortality [8, 13]. Moreover, prior reports have conceded THA is associated with increased complications in the treated individuals [11, 24]. Early prosthesis revision is acknowledged as a key adverse event and has been the focus of several reports related to THA [10]. Although a recently published assessment of CTHA and UTHA demonstrated improved safety profiles with UTHA, some of the studies have a relatively small sample size, which renders their conclusions unconvincing [22, 25]. Although our study focuses on the early stages of follow-up and UTHA appears to be undifferentiated from CTHA regarding HHSs and complication rates, it will be of great consequence to determine whether resistance to bone microstructure occurs in the later stages of follow-up and, if so, by what mechanism [12, 25].

Two recent meta-analyses indicated that CTHA was as effective, if not more so, than UTHA [26, 27]. The improvement in HHS with CTHA was important, particularly since there were fewer early prosthetic revisions with CTHA than with UTHA [28, 29]. Although a high complication rate and poor quality of life were associated with UTHA [30], a rapid recovery process was observed, without significant differences in the HHSs at the first or second month after surgery. Because of the increasing complication rates, the ideal treatment strategy for femoral fracture is of concern. Prior randomised controlled studies [25, 31] with long-term follow-up demonstrated that UTHA had an influence on the treatment outcomes with regards to the increased incidence of complications, such as loosening of prostheses. Despite the clear benefit of CTHA, a growing, but still very limited body of literature, has shown a distinctly higher rate of early revision after CTHA treatment among patients in some other medical centres [14, 32].

There has always been an argument about the use of uncemented versus cemented femoral components in THA [33]. Although UTHA and CTHA have gained enormous popularity in recent decades, experiencing rapid adoption, there remains no consensus on which device should be used for femoral neck fractures, and it could be a long time before consensus is reached [34, 35]. Furthermore, the evidence based on the latest reports [22, 36] regarding whether initial UTHA or CTHA is the optimal strategy is unclear. The principles behind CTHA and the low friction of metal-polyethylene are extremely important for the modification of joint surgery [7]. However, long-term loosening is commonly deemed to be the main cause of CTHA failure [8, 12]. To date, CTHA remains considered the gold standard for assessing other prosthetic surgeries, and early weight-bearing is easier to achieve following CTHA than following UTHA [8, 9].

Several limitations should be acknowledged. First, selection bias seems impossible to avoid because a large number of patients were excluded. Furthermore, residual bias from confounding factors remains because some information was unavailable and therefore was excluded. Second, our analysis is a retrospective observational study with some problems inherent to the methodology. Confounders due to the research methods may have reduced the power to draw reliable conclusions, but well-matched cohorts rendered us able to draw conclusions that were irrespective of the baseline data. The purpose of our study was to compare the long-term clinical outcomes for patients with femoral neck fracture (AO/OTA type 31B) treated with primary unilateral UTHA or CTHA. It is important to emphasise that we cannot provide a comprehensive recommendation to further improve outcomes for all individuals undergoing CTHA based on the current results.

## Conclusions

The results reported in this study support a growing body of evidence that CTHA is superior to UTHA in an Asian population with femoral neck fracture (AO/OTA type 31B). To date, controversy remains about the use of CTHA or UTHA, and the type of surgical approach used should be fully evaluated, along with the patient’s age, activity level, and life expectancy, with the aim of achieving the optimal clinical efficacy.

## Data Availability

The datasets used and/or analysed during the current study are available from the corresponding author upon reasonable request.

## Abbreviations

AP: Antero-posterior
ASA: American Society of Anesthesiologists
BMD: Bone mineral density
BMI: Body mass index
CTHA: Cemented femoral component total hip arthroplasty
DAA: Direct anterior approach
HHS: Harris hip score
ISS: Injury severity score
SD: Standard deviation
UTHA: Uncemented femoral component total hip arthroplasty
